# Dual Sound Sources in Siamangs Generate Individually Rhythmic and Temporally Coordinated Vocal Emissions

**DOI:** 10.1111/nyas.70273

**Published:** 2026-04-21

**Authors:** Lia Laffi, Alice Salerno, Olivier Friard, Silvia Poletti, Giorgia Ruffa, Martina Tubito, Vittorio Luigi Bianco, Daria Valente, Michele Capasso, Roberta Castiglioni, Valentina Isaja, Andrea Ravignani, Marco Gamba

**Affiliations:** ^1^ Department of Life Sciences and Systems Biology University of Turin Turin Italy; ^2^ Fondazione ZOOM Cumiana Italy; ^3^ Department of Veterinary Medicine and Animal Production University of Naples Federico II Naples Italy; ^4^ DARWIN Ricerca e Divulgazione Naturalistica Milano Italy; ^5^ Department of Human Neurosciences Sapienza University of Rome Rome Italy; ^6^ Centre for Music in the Brain, Department of Clinical Medicine Aarhus University & The Royal Academy of Music Aarhus/Aalborg Denmark; ^7^ Research Center of Neuroscience “CRiN‐Daniel Bovet” Sapienza University of Rome Rome Italy; ^8^ Institute of Cognitive Sciences and Technologies, National Research Council Rome Italy

**Keywords:** isochrony, laryngeal sac, multimodality, rhythmic categories, singing primate

## Abstract

Temporal organization is crucial for efficient communication in many animal species, particularly those with complex vocal output. Singing primates structure their songs into rhythmic categories based on small‐integer ratios between adjacent intervals. However, rhythmic organization usually emerges from a single sound source, and it remains unknown whether any species can produce rhythms through the coordination of multiple vocal production mechanisms. This study investigates rhythmicity in the song of siamangs (*Symphalangus syndactylus*). This singing primate uses two distinct vocal mechanisms: modulation of the oral tract and radiation through the laryngeal sac. We analyzed the temporal structure of vocal units resonated in the oral tract (mouth units) and those by the laryngeal sac (sac units), as well as the combined output of both. Both mouth and sac units displayed an isochronous pattern (1:1), corresponding to adjacent intervals of the same duration. The combined output showed significant isochrony (1:1) and two additional nonsignificant clusters at ratios of 1:9 and 9:1. These findings suggest that the interaction between the two vocal mechanisms yields a more complex rhythmic structure, representing a unique case of vocal coordination in nonhuman primates.

## Graphical abstract

We analyzed the rhythmic structure of songs from 14 adult siamangs, a singing primate that produces units via two distinct vocal production mechanisms: oral‐tract modulation and laryngeal‐sac resonance. When considered separately, both mechanisms exhibit isochrony (1:1). In contrast, the combined output reveals additional rhythmic clusters (≈1:9, 9:1). This dual‐source mechanism generates complex rhythmic patterns, offering a unique case of multimodal vocal rhythmicity in nonhuman primates.

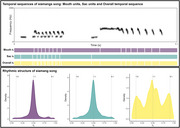

## 1 Introduction

Rhythmicity represents a fundamental organizing principle of biological systems and animal life. In the behavioral domain, temporal regularities are particularly relevant in communicative signaling. In the last few years, increasing evidence has shown that different taxa temporally organize vocal and nonvocal signals in rhythmic categories, arising from repeated relationships between adjacent intervals, usually small‐integer ratios (e.g., 1:1, 1:2) (e.g., bats [1], primates [2, 3, 4, 5, 6], rock hyraxes [7], seals [8, 9], insects [10], fishes [11], and birds [12, 13]). Interestingly, all these studies focus on the rhythmicity of unimodal signals, but communication often relies on multiple integrated signals to convey information [14, 15]. Multimodal communication is itself characterized by rhythmicity, emerging from the temporal alignment and coordination of signals produced across different modalities [16], either through synchrony [17], or sequential coordination across modalities [18]. To our knowledge, research on animal vocal rhythmicity has so far focused almost exclusively on unimodal signals, and the rhythmic organization of multiple vocal production mechanisms remains unexplored.

The comparative framework emerging from the quantification of rhythmic categories in different taxa might be a key tool to understand the evolution of rhythm in vocal communication. Particularly relevant for this purpose are singing primates, a nonmonophyletic group of species producing hierarchically organized sequences of frequency‐modulated units, namely, songs [19]. Previous studies on singing primates’ songs have evidenced the production of rhythmic categories that are coherent with those emerging in human music. Coppery titi monkeys (*Plecturocebus cupreus*) produce adjacent intervals of the same duration, corresponding to local isochrony (1:1 rhythmic category) [20]. Similarly, the lemur *Indri indri* performs songs characterized by local isochrony and two additional rhythmic categories [21]. Among gibbons, lar and crested gibbons (*Hylobates lar*, *Nomascus gabriellae, Nomascus leucogenys, Nomascus siki, Nomascus nasutus, Nomascus hainanus*, and *Nomascus concolor*) display an isochronous temporal organization with temporal adjustment to facilitate coordination [5, 22, 23]. Notably, *N. hainanus* exhibits an additional rhythmic category based on a 2:1 ratio and a rhythmic category not corresponding to a small‐integer ratio [23]. Collectively, all these findings suggest that regular timing patterns characterize the production of complex vocal sequences in singing primates and may aid vocal coordination during duetting. Here, we extend this framework by investigating rhythmicity in siamangs (*Symphalangus syndactylus*), a singing primate species characterized by a unique dual vocal production system, thereby offering the opportunity to examine rhythmic organization arising from multiple vocal modalities.

**FIGURE 1:**
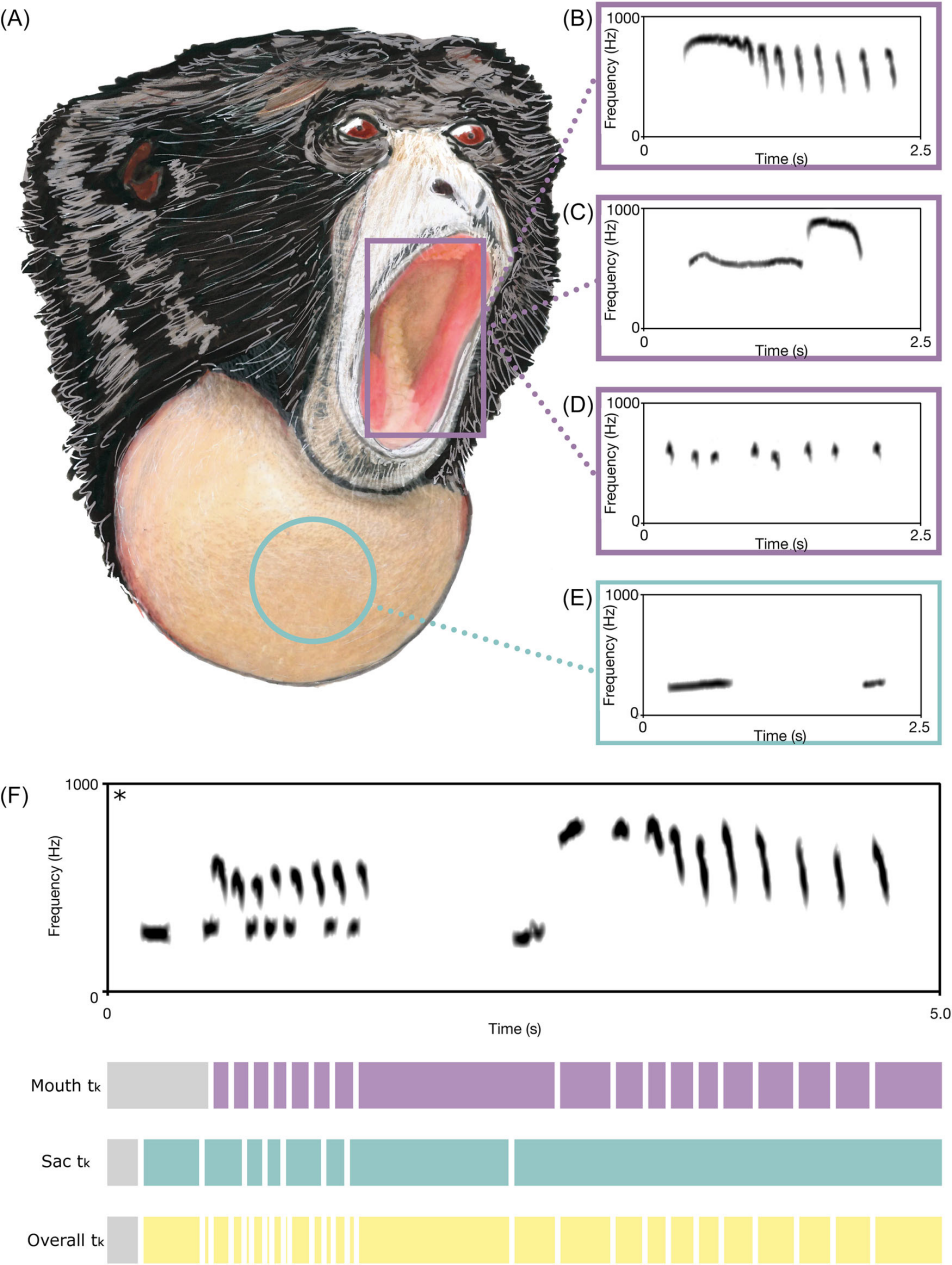
Siamang vocalizations and acoustic feature extraction. (A) A sketched siamang (*Symphalangus syndactylus*) while singing. (B–E) Spectrograms of representative unit types produced through mouth resonation, or radiated through the laryngeal sac: (B) ululating scream, (C) bitonal scream, (D) barks, and (E) booms. (F) Spectrogram and sequence of intervals (*t*_*k*_) of mouth units (*purple*), sac units (*light blue*), and overall acoustic output (*yellow*). All spectrograms are generated in Praat with the following parameters: window length: 0.025 s; time range as shown; frequency range: 0–1000 Hz; maximum: 100 dB/Hz; dynamic range: 35–45 dB; pre‐emphasis: 6 dB/oct; dynamic compression: 0. * denotes a different temporal scale from the previous spectrograms.

Siamangs (Figure 1A) live in small, territorial groups and produce loud songs that can be heard over long distances [24, 25]. Although air sacs are present in other gibbons and across primates [26], siamangs have particularly large and visible laryngeal air sacs in both males and females. The air sac acts as a resonating structure, enhancing vocal output and enabling the production of high‐intensity, low‐frequency vocalizations, *boom* units [27, 28]. This anatomical peculiarity enables a specialized phonatory mechanism characterized by two distinct resonating pathways, corresponding to two putative vocal sources. Specifically, booms are radiated through the vocal sac, and are emitted while maintaining the mouth closed [27]; all the other units (*barks*, *ululating screams*, and *bitonal screams*) resonate in the mouth during the song and are shaped by the vocal tract [29, 30]. Although units radiated through the laryngeal air sac and mouth‐open units involve distinct resonating pathways, they do not constitute independent vocal production mechanisms. Previous work has shown a strong temporal coupling between the two, with booms typically preceding mouth units [31, 32], and a strong functional coupling, as the air sac inflation during booms is associated with spectral characteristics of the following barks [29]. Given this unique dual vocal production system, it becomes particularly interesting to investigate whether the rhythmic structure of siamang songs is shared with other gibbon species or represents a species‐specific adaptation. Are rhythmic categories differentially associated with vocal units radiated through the air sac versus those modulated by the oral tract? What is the temporal organization of the song? First, we ask whether each vocal mechanism independently produces a rhythmic structure. Second, we investigate how the two mechanisms interact and whether their coordination generates a joint rhythmic organization.

This study analyzes a unique case of a nonhuman primate vocal system that integrates two distinct sound generation mechanisms, potentially providing new insights into the vocal rhythmicity of singing primates. Here, we quantify the temporal organization of siamang songs from 14 adult individuals and identify rhythmic categories within unit sequences. Specifically, we test: (1) the temporal patterns of units produced with the mouth open and modulated by the oral tract; (2) the sequences of units radiated through the vocal sac and produced with the mouth closed and an inflated laryngeal sac; and (3) the combined vocal output including all units, both modulated by the oral tract and radiated by the laryngeal sac. Our first prediction is that the sequence of mouth‐open emitted units displays an isochronous pattern, similar to those of lar and crested gibbons [5, 22, 23]. Our second prediction is that the time series of sac units will exhibit a temporal structure similar to the mouth units, given the strong temporal coordination and functional coupling of sac and mouth units [25, 29]. Our third prediction is that the overall vocal output will exhibit a rhythmic structure that differs from those observed for each phonatory mechanism considered alone. Specifically, if we expect that both mechanisms produce isochronous sequences, their combination is unlikely to result in a similarly isochronous pattern unless the two sequences are tightly synchronized or occur in antiphase. For this reason, we expect the emerging rhythm to differ in structure from its components [16].

## 2 Materials and Methods

### 2.1 Study Species and Song Structure

Siamangs live in small, territorial, often multimale groups comprising adults and their offspring [32, 33]. Interestingly, they are highly vocal and produce choruses mainly related to pair bonding and territorial defense [34]. Siamang's song has been described as one of the longest, lasting up to 30 min, and among the most complex vocal outputs in nonhuman primates [31]. Those high‐intensity vocalizations are essential for long‐distance communication [28, 35]. Songs are hierarchically organized: sequences of units are organized in phrases, and groups of phrases compose sequences [31].

Songs may feature various types of vocalizations [31]. The booms are low‐frequency units emitted when the sac is inflated, and the mouth is closed (Figure 1E). Barks, together with booms, are the most frequent type of vocalization in the siamang song (Figure 1D). A short boom usually precedes a bark, and the pair of units may be emitted either in a rapid series of boom−bark sequences or as an isolated pair. Ululating screams are long units of slightly decreasing frequency (Figure 1B). Booms usually precede them and are followed by a sequence of short, fast barks, with a decelerando. The last vocal type is the bitonal scream, which consists of two phases that can be emitted consecutively or separated by a silence (Figure 1C). The first phase is typically long and exhibits low‐frequency modulation, while the second phase is shorter and more modulated. Barks, ululating screams, and bitonal screams are all produced with an open mouth. Both males and females may emit all these units, except for the bitonal scream, which is exclusive to males [31].

The structure of a typical siamang song follows a predictable pattern, but with a certain degree of flexibility [36, 37]. The song structure has been extensively described by Geissmann [30]. The song typically begins with an introductory sequence that often features short single barks and brief series of barks, accompanied by short booms. The interlude sequence is highly variable, with a flexible combination of booms, barks, and ululating screams. A series of booms usually ends the interlude sequence, marking the beginning of the great call sequence. The great call sequence includes all types of vocalizations. After the sequence of booms that concludes the interlude sequence, the female emits a series of accelerated barks, characterized by a shortening of barks, of booms, and of silences between units. At the peak of the female acceleration, the male emits a bitonal scream. The female emits a second series of accelerated barks, and the male a series of short, fast barks followed by an ululating scream. As the vocal tempo increases further, the female emits an ululating scream and joins the male in a coordinated sequence of short, fast barks [31]. The last part of the sequence is characterized by the animals’ movement during the vocal display, with amplitude increasing with body acceleration [38].

### 2.2 Dataset

The complete dataset comprises recordings from seven zoos, featuring 14 adult individuals. We recorded songs in the form of solo songs, duets, or choruses [19] at three zoos: ZOOM Torino, Parco Faunistico Le Cornelle, and Safari Ravenna (Table S1). At ZOOM Torino, 26 recordings were collected between September 7, 2011 and February 28, 2025, using various recorders (Sennheiser ME66 with K3U, Tascam DR‐40, AudioMoth). At Parco Faunistico Le Cornelle, 13 recordings were made between October 19, 2020 and December 6, 2020, using an AudioMoth recorder. At Safari Ravenna, 56 recordings were gathered between October 12, 2024 and December 7, 2024, also using an AudioMoth recorder. Having collected all sounds opportunistically, each recording may contain either a complete or a partial song. The AudioMoth recorder followed a sampling schedule, recording for 59.5 min every hour, which allowed for near‐continuous recording from 08:00 to 17:00. To increase our sample size, we also included audio files sourced from YouTube. We included five recordings of the dominant couple at the Cincinnati Zoo, 10 from the Dubbo Zoo, two from the Houston Zoo, and five from the Miami Zoo. Audio files sourced from YouTube consist of partial songs. Overall, individuals are unevenly represented in the dataset (Table S1), and recording methods and sampling protocols vary across sites and sources. However, this dataset includes vocal recordings collected across different locations and seasons, providing a representative sample of siamang singing behavior.

### 2.3 Acoustic Analyses

We edited the songs as WAV audio files using the software Praat 6.0.56 [39]. We used Praat *TextGrid* to annotate onsets and offsets of each unit for each individual contributing to the song. Using different recording devices does not affect the annotation, as our analyses focus solely on the timing of vocal units rather than their spectral properties: in all cases, the annotation of units was made when onsets and offsets were clearly visible. We created two annotation tiers for each individual: one for units emitted with the mouth open and modulated by the oral cavity (hereafter, mouth units), and one for units radiated through the vocal sac, with the mouth closed (hereafter, sac units). For each individual, we extracted the onsets for both tiers (mouth and sac units) from the *TextGrid* files and exported them into a table using a Python script [40].

We analyzed the rhythmic structure of three different temporal sequences: (1) the first analysis focused on mouth units only (hereafter, mouth sequence); (2) the second analysis focused on the sac units only (hereafter, sac sequence); (3) the third one focused on the overall output at the individual level, considering all the units produced by an individual, regardless of the mechanism of vocal production (hereafter, overall output) (Figure 1F).

Using R 4.2.1 [41], we calculated the inter‐onset intervals (*t*_*k*_) as the interval between two consecutive unit onsets. To remove potential outliers, we selected *t*_*k*_ < 5 s to be consistent with available data on other primates [5]. We then calculated the rhythmic ratio (*r*_*k*_) by dividing each *t*_*k*_ by its duration plus the duration of the following interval [42]: *r*_*k*_ = *t*_*k*_ /(*t*_*k*_ + *t*_*k+1*_).

### 2.4 Statistical Analyses

#### 2.4.1 Defining Rhythmic Categories

We tested whether *r*_*k*_ significantly fell around small‐integer ratios using a previously published methodology [42]. We tested the presence of the 1:1 rhythmic category, corresponding to local isochrony, across all temporal sequences, mouth and sac sequences, and the overall output. After visual inspection of the *r*_*k*_ density distribution, we also tested two additional rhythmic categories, 1:9 and 9:1, in the overall output. For each tested category (1:9, 1:1, and 9:1), we defined an on‐integer ratio range centered around a small‐integer ratio, and two adjacent off‐integer ratio ranges (see Supplementary Materials—“On‐integer and off‐integer ratio ranges”). We then counted the number of *r*_*k*_ datapoints falling within each on‐ and off‐integer ratio range per individual contribution (hereafter, *r*_*k*_ count).

#### 2.4.2 Data Normalization

As the on‐integer and off‐integer bins differ in width, empirical *r*_*k*_ counts are not directly comparable, as wider bins would be expected to contain more observations by chance. Various normalization methods have been proposed in prior research, and we decided to use a method proposed and described by Jadoul and colleagues [43]. Contrary to previous methods, which normalize *r*_*k*_ counts assuming a uniform distribution of *r*_*k*_ values (the *r*_*k*_ null distribution [42]), we derived the *r*_*k*_ null distribution from the most random possible distribution of *t*_*k*_ values. Since the range of inter‐onset intervals emitted is constrained by physiological factors, this methodology assumes that the null distribution of *t*_*k*_ is uniform between the minimum and maximum values observed in the empirical data. Following the Jadoul methodology, we calculated a uniform *t*_*k*_ distribution over the minimum and maximum inter‐onset interval values observed in the empirical distributions of mouth sequence, sac sequence, and overall output. We calculated *r*_*k*_ values from the *t*_*k*_ null distributions, then determined the expected probability for each bin as the *r*_*k*_ count in that bin. The expected probability was used to normalize the empirical *r*_*k*_ counts. This process was iterated three times separately for the mouth sequence, sac sequence, and overall distribution, as the minimum and maximum *t*_*k*_ values vary across the empirical *t*_*k*_ distributions.

#### 2.4.3 Testing Rhythmic Categories

We used generalized linear mixed models (GLMMs) to test whether siamangs produced significantly more on‐integer than off‐integer ratios for 1:1, 1:9, and 9:1 rhythmic categories (*glmmTMB* package [44]). For each rhythmic category, we initially fitted two models: one with and one without sex as a fixed effect. As model comparisons based on the Akaike Information Criterion consistently favored simpler models without sex, we excluded it from all models. We ran three GLMMs, one for each rhythmic category, fitting a negative binomial distribution. For all models, the negative binomial distribution was selected over the Poisson distribution to account for overdispersion in the data (*performance* package [45]). Moreover, we checked models to exclude zero inflation (*performance* package). All models included the *r*_*k*_ counts as a response variable and individual identity as a random factor. The fixed factor changed across models.

- In the first model, we included the particular bin in which the *r*_*k*_ fell (on 1:1, off 1:1) and the type of unit sequence (mouth sequence, sac sequence, overall output) as fixed factors.
- The second model tested the significance of the 1:9 rhythmic category. We tested rhythmic categories uniquely for the overall output. We included the bin in which the *r*_*k*_ fell (on 1:9 and off 1:9) as a fixed factor.
- The third model tested the significance of the 9:1 rhythmic category. As with the second model, we tested rhythmic categories only on the overall output. We included the bin in which the *r*_*k*_ fell (on 9:1 and off 9:1) as a fixed factor.

Since the on‐integer and off‐integer ratio ranges have uneven widths, we also included an offset variable for the three models that weights the *r*_*k*_ count based on the expected probability previously calculated, as explained in the previous paragraph. To test the significance of the three full models, we compared them with null models that included only the random factor and the offset, using a likelihood ratio test [46]. We obtained *p*‐values for each predictor (R *summary* function) and pairwise comparisons (*emmeans* package [47]). We checked normality and homogeneity of residuals by inspecting the distribution of residuals and the *qqplot* (a function provided by R. Mundry).

#### 2.4.4 Unit Transition Contribution to the General Isochrony

The overall vocal output was composed of mouth and sac units, defining a sequence of *t*_*k*_. To analyze this sequence, we classified each *r*_*k*_ based on the anatomical vocal mechanism of the two *t*_*k*_ used to calculate it, as well as the vocal mechanism of the following unit. For example, within a sequence of two following mouth units, the resulting *r*_*k*_ was classified as mouth‐mouth‐mouth when the two units were followed by a third mouth unit, or mouth‐mouth‐sac if the third unit was produced with the sac. We thus identified eight possible categories (*r*_*k*_ type): mouth‐mouth‐mouth, mouth‐mouth‐sac, mouth‐sac‐mouth, mouth‐sac‐sac, sac‐mouth‐mouth, sac‐mouth‐sac, sac‐sac‐mouth, sac‐sac‐sac. We analyzed whether there were differences in the isochronous production for these categories. We classified each *r*_*k*_ of the overall output as on‐integer (0.444 < *r*_*k*_ < 0.556) or off‐integer (0.400 < *r*_*k*_ < 0.444, or 0.556 < *r*_*k*_ < 0.600) and counted the number of on‐ and off‐integer *r*_*k*_ for each category within each recording. To avoid zero inflation, we excluded categories with no observations in either the on‐integer or off‐integer counts. We modeled the counts of on‐ versus off‐integer *r*_*k*_ using a GLMM (*glmmTMB* package) with a binomial distribution, with the response representing the number of successes (on‐integer) and off‐integer counts treated as failures. We imposed the *r*_*k*_ type as a fixed factor, and individual identity and recording as random factors. We obtained *p*‐values for each predictor (R *summary* function) and performed pairwise comparisons (*emmeans* package).

#### 2.4.5 Visualizations of the Temporal Organization of Different Transition Types

To examine the fine‐scale timing of vocal events, we created a jitter plot showing the distribution of rhythmic ratios (*r*_*k*_) across eight types of transitions. Each *r*_*k*_ was calculated from the onset times of two adjacent units (*t*_*k*_), allowing us to categorize each *r*_*k*_ according to the production mechanism (sac vs. mouth). The onset of the third following unit was used only to identify the type of transition for categorization. This method allowed us to more precisely group temporal intervals by considering the production mechanism of three adjacent units: the units used to compute the *r*_*k*_ and the following one.

## 3 Results

### 3.1 Descriptive Statistics of the *t*_*k*_ and *r*_*k*_ Distributions

**FIGURE 2:**
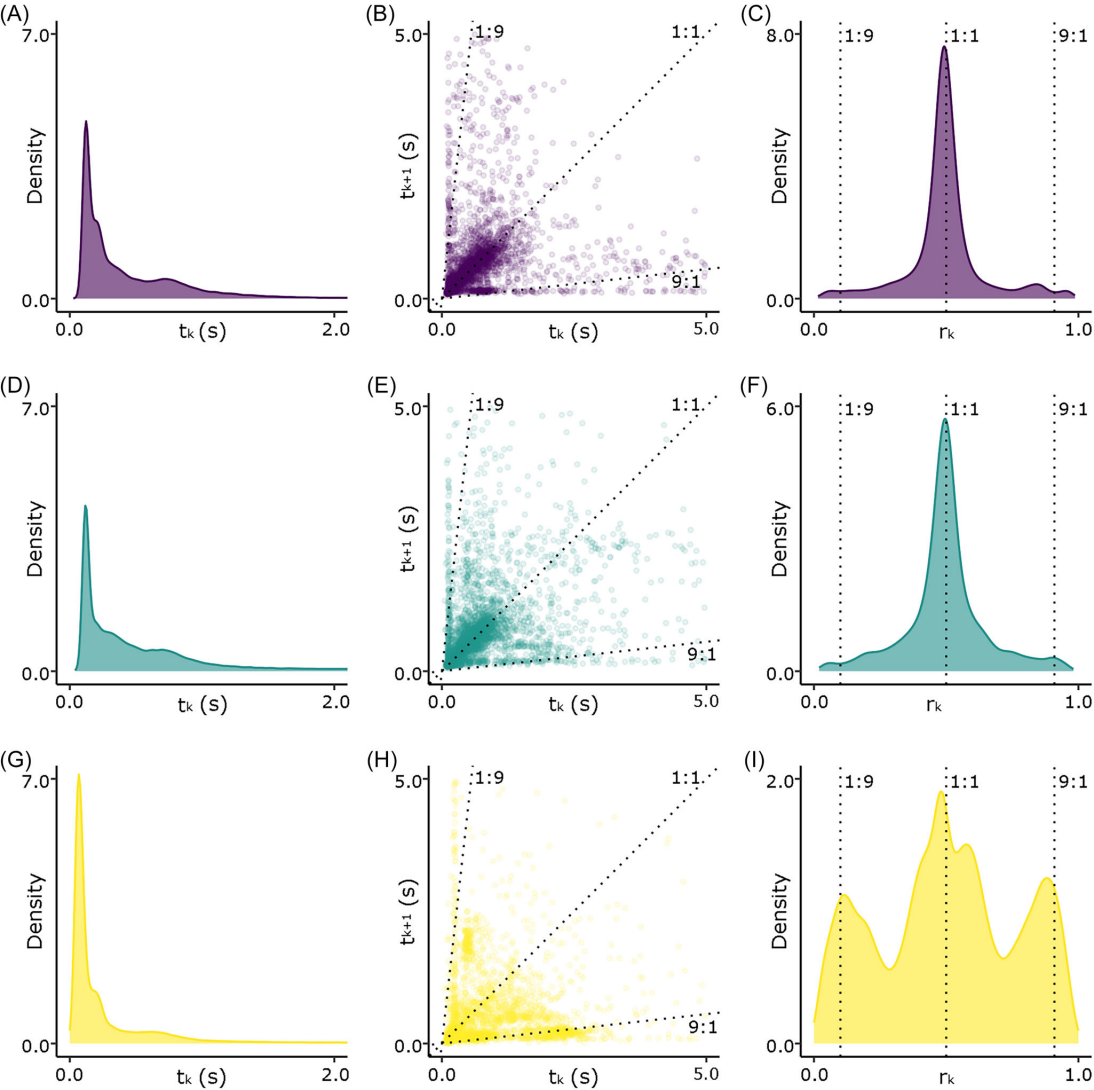
Rhythmic representation of siamang's songs: purple plots refer to the sequence of units produced by the mouth; light blue plots refer to units produced by the sac; yellow plots refer to the overall vocal output (A, D, G). Density plot of *t*_*k*_ (B, E, H). Transition scatter plot representing the relationship of adjacent intervals. Each point represents a pair of consecutive time intervals. Density plot of rhythmic ratios (*r*_*k*_) (C, F, I).

The distribution of the inter‐onset intervals shows a single peak for either mouth units, sac units, and overall contribution (Figure 2A,D,G). We found *t*_*k*_ peak values of 0.125, 0.130, and 0.069 s in the mouth sequence, sac sequence, and overall output, respectively. The *r*_*k*_ distribution for both mouth and sac units is unimodal with a peak value of 0.492 for mouth and 0.497 for sac units. The overall output of the *r*_*k*_ distribution shows three distinct peaks at 0.115, 0.481, and 0.880 (Figure 2C,F,I).

### 3.2 Testing Rhythmic Categories

We tested whether siamangs produced significantly more on‐integer than off‐integer rhythmic ratios around the 1:1 category in mouth and sac sequences, as well as around the 1:1, 1:9, and 9:1 categories in their overall output, where both sac and mouth units were considered. We found that the mouth and sac sequences, as well as the overall output, show an isochronous pattern (Table 1). The two GLMMs testing for 1:9 and 9:1 rhythmic categories showed no significant differences relative to the null models, suggesting that neither 1:9 nor 9:1 was significant.

**TABLE 1:** Generalized linear mixed model (GLMM) results.

GLMM	Full versus null			Post‐hoc comparisons				
	Chisq	Df	*p*	Contrast	Estimate	SE	Z ratio	*p*
**1:1 rhythmic category**	416.727	5	<0.001	Overall output	−0.321	0.092	−3.493	0.006
				Mouth units	−1.526	0.093	−16.335	<0.0001
				Sac units	−1.175	0.093	−12.660	<0.0001
**1:9 rhythmic category**	0.144	1	0.705					
**9:1 rhythmic category**	0.167	1	0.683					
**Units transition contribution to isochrony**	7029.300	7	<0.001	m‐m‐m/m‐m‐s	10.410	0.756	32.244	<0.0001
				m‐m‐m/m‐s‐m	12.825	0.512	63.891	<0.0001
				m‐m‐m/m‐s‐s	8.078	0.522	32.315	<0.0001
				m‐m‐m/s‐m‐m	15.020	2.050	19.839	<0.0001
				m‐m‐m/s‐m‐s	12.102	0.483	62.460	<0.0001
				m‐m‐m/s‐s‐m	11.421	2.540	10.942	<0.0001
				m‐m‐m/s‐s‐s	7.578	0.639	24.005	<0.0001
				m‐m‐s/m‐s‐m	1.232	0.080	3.216	0.028
				m‐m‐s/m‐s‐s	0.776	0.064	−3.078	0.043
				m‐m‐s/s‐s‐s	0.728	0.072	−3.230	0.027
				m‐s‐m/m‐s‐s	0.630	0.034	−8.565	<0.0001
				m‐s‐m/s‐m‐s	0.944	0.017	−3.287	0.023
				m‐s‐m/s‐s‐s	0.591	0.045	−6.866	<0.0001
				m‐s‐s/s‐m‐m	1.859	0.264	4.368	<0.001
				m‐s‐s/s‐m‐s	1.498	0.080	7.494	<0.0001

*.Note*: Only statistically significant comparisons are reported. In contrast, “mouth” and “sac” are abbreviated as “m” and “s,” respectively.

### 3.3 Units Transition Contribution to the General Isochrony

The model suggests that the different types of *r*_*k*_, identified by the vocal mechanisms of emission of the two units generating the *r*_*k*_ and the following unit, show notable differences in rhythmic structure (Table 1). In particular, mouth‐mouth‐mouth unit sequences display a much stronger tendency toward isochrony, and thus a higher production of 1:1 on‐integer *r*_*k*_ compared to off‐integer ones, than all other types.

### 3.4 Temporal Organization of Different Transition Types

**FIGURE 3:**
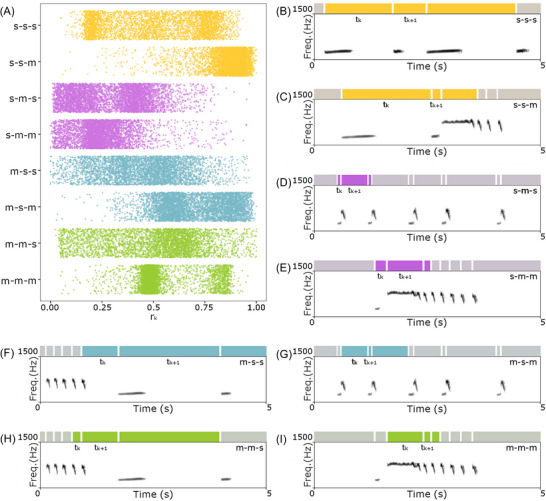
Rhythmic transitions within siamang vocal sequences. (A) Jitter plot of rhythmic ratios (*r*_*k*_) across different transition types, revealing distinct temporal organization patterns. Transition labels: m = mouth unit; s = sac unit. (B−I) Explanatory spectrograms of different transition types: colored bars report the *t*_*k*_ and *t*_*k+1*_ corresponding to units used to calculate the *r*_*k*_. Each color indicates a transition between two unit typologies (m or s), illustrated in two cases depending on whether the subsequent unit is a mouth or a sac unit. Yellow plots refer to the transition of two sac units (s‐s); purple plots refer to a sac unit followed by a mouth unit (s‐m); light blue plots refer to a mouth unit followed by a sac unit (m‐s); green plots refer to the transition between two mouth units (m‐m). All spectrograms are generated in Praat with the following parameters: window length: 0.035 s; time range as shown; frequency range: 0–1500 Hz; dynamic range: 60 dB.

The jitter plot reveals that different transitions exhibit distinct temporal patterns, which can be linked to specific parts of song sequences (Figure 3A). The sac‐sac transitions followed by sac unit (sac‐sac‐sac) displayed a bimodal distribution with two distinct clusters of *r*_*k*_ values. This pattern is compatible with the production of boom sequences, as shown in Figure 3B, which displays a boom series typically found at the end of the interlude sequence. In sac‐sac‐mouth transitions, a concentration of points can be observed around the 9:1 rhythmic category. An example of this transition is shown in Figure 3C, in which a ululating scream follows two consecutive booms. This pattern can be observed in the interlude sequence.

Sac‐mouth transitions followed by sac unit (sac‐mouth‐sac) also showed two clusters, with a cluster shifted toward the left of perfect isochrony, and a secondary peak close to the 1:9 rhythmic category. This pattern is consistent with boom−bark sequences. Specifically, the 1:9 pattern is consistent with an isolated bark preceded by a boom (Figure 3D), and the isochronous pattern with a short, fast sequence of barks alternated with booms. Sac‐mouth‐mouth transitions were more dispersed but showed a tendency toward a single cluster of points. This pattern can be related to an ululating scream and its preceding boom. An example is shown in Figure 3E, which displays the typical sequence of an ululating scream followed by short, fast barks.

Mouth‐sac‐sac transitions exhibited a broader distribution, with no sharp clustering. An example is shown in Figure 3F, which captures the end of a bark series following an ululating scream and the beginning of a new boom sequence. Mouth‐sac‐mouth transitions show a bimodal distribution, with one cluster around isochrony and a second cluster closer to the 9:1 rhythmic category. The first peak appeared slightly shifted to the right compared to perfect isochrony. This pattern can be related to sequences of single barks or fast bark sequences preceded by booms. An example of this transition appears in Figure 3G, where a sequence of bark is shown.

Mouth‐mouth‐sac transitions do not show clear peaks; an example of this transition is shown in Figure 3H. The mouth‐mouth‐mouth transitions exhibited strong clustering around isochrony, suggesting similar durations between consecutive mouth intervals. This pattern emerges from the series of short barks typically emitted after the ululating screams. A second cluster close to the 9:1 rhythmic category is compatible with the transition from the ululating scream to the first following bark (Figure 3I).

These findings suggest that the isochronous pattern of siamangs’ song is primarily associated with specific parts of the unit sequences, namely, the long sequences of rapid booms and barks, as well as the sequence of barks following ululating screams. On the other side, the cluster of rhythmic ratios near 1:9 and 9:1 can be linked to isolated barks preceded by a boom, to sequences of shorter and longer booms, and ululating screams.

## 4 Discussion

We found that the siamang songs are temporally organized. Specifically, the two mechanisms of vocal production each generate an isochronous sequence, and the overall vocal output produces a new collective rhythmic output. We also found that different unit transitions contribute differently to the overall rhythmic structure.

Similar to other gibbons and consistent with our first prediction, we found that the sequence of units emitted with an open mouth and modulated by the oral tract (bark, bitonal screams, ululating screams [30]) is locally isochronous. Isochrony derives from the succession of equal‐duration intervals and is the simplest rhythmic structure to describe. In vocal emissions, isochrony has been proposed to have three primary adaptive functions: enhancing signal transmission by increasing its predictability, facilitating coordination among vocalizers, and signaling mate quality [48]. Indeed, siamang choruses play a key role in long‐distance communication between different groups [35]. In this sense, isochrony would facilitate signal transmission in the highly dense environments where siamangs live [49]. Second, in other singing primates, isochrony is important for coordination among the different individuals contributing to the chorus, and this function is strongly supported in other gibbon species, which are the closest taxonomic group to siamangs [22, 23]. Third, we cannot rule out the possibility that the isochrony of vocal output plays a role in signaling partner quality, as documented in other gibbons species, both phylogenetically and ecologically close to siamangs [5, 23]. Of course, disentangling the evolutionary drivers shaping siamangs’ rhythmicity could be challenging. Yet, the three factors mentioned above may all have contributed to the development of the isochronous temporal organization of siamang songs throughout their evolutionary history.

Our results confirm our second prediction: units produced through radiation from the laryngeal sac, boom units [27], indeed exhibit a similar temporal organization to units produced with the mouth open. Rather than serving as isolated vocal elements, booms often serve as introductory units for barks, bitonal screams, and ululating screams [31]. In particular, most booms immediately precede barks, and the air sac inflation is related to the spectral feature of the following mouth unit [29]. The strong functional and temporal coupling between booms and subsequent units aligns with a similar rhythmic structure in laryngeal sac−radiated and oral tract modulated units.

Consistently with our third prediction, the temporal organization of the overall output differs from that of the two vocal mechanisms considered separately. The mouth and sac sequences resemble an isochronous sequence in which all intervals have similar durations. In contrast, the distribution of the overall output qualitatively reveals the presence of local isochrony (1:1 rhythmic category), as well as an alternation between long and short intervals, which is reflected in the two additional clusters of rhythmic ratios near 1:9 and 9:1. However, these rhythmic categories do not reach statistical significance [42]. The emergence of an isochronous rhythmic organization in the overall output, similar to that of the two modalities when considered alone, is an interesting result and far from being obvious. The overall output is composed of parts characterized either by the emission of a single source, as sequences of booms or an ululating scream followed by barks, or by the combined use of the two sources. For sequences emitted by the same source, an isochronous sequence is expected. However, an isochronous collective rhythm can emerge from two isochronous sources with similar interval duration only in two cases: the mouth and sac units can be emitted in phase, with a synchronized emission in the two sources, or in antiphase, with a lag of emission of half the interval duration between the two sources. Our results show that local isochrony is present in sequences based on the alternation of mouth and sac units (Figure 3A), suggesting that the isochrony of the collective rhythm is, therefore, also influenced by the antiphase emission of mouth and sac units in rapid boom−bark sequences. The two additional clusters near the 1:9 and 9:1 also arise from specific structures in the song characterized by the alternation of mouth and sac units. Specifically, we can find this temporal structure in sequences of isolated boom−bark units (Figure 3G), which generate systematic alternations between long and short intervals. Overall, the rhythmic structure emerging from the emission of sac and mouth units can be explained by the strong temporal coupling between boom and bark units, which are the most frequently represented in the song [25, 31]. Indeed, the presence of rapid boom−bark sequences is consistent with the emission of local isochrony, whereas the emission of boom−bark pairs that are more widely spaced in time accounts for the presence of the two additional clusters (Figure 2H).

Overall, the presence of three rhythmic categories in the collective rhythmic structure of siamang song suggests that these categories constitute characteristic features of siamang song organization. As in other gibbons, an isochronous pattern in the overall output might improve the coordination of adults in nonmonogamous mating systems [23]. In addition, isochrony at the level of the overall output may also play a role in mate quality signaling, as maintaining an isochronous structure despite continuous switching between two distinct vocal production sources may reflect constraints on motor control and vocal coordination [7]. However, the temporal organization of siamang song is a unique rhythmic output among gibbons, reflecting a complex multimodal rhythmicity that emerges from a dual mechanism of vocal production [5, 22, 23].

Anatomy may affect rhythmic patterning as well, though other factors are likely involved. Across primates, the simplification of laryngeal anatomy and the reduction of vocal membranes and the laryngeal sac are associated with more stable phonation and a decrease in nonlinear phenomena and chaos [50, 51]. Despite anatomical variations, studies on the temporal structure of vocalizations across species—including siamangs, indris, titi monkeys, and humans—reveal a common tendency to produce sequences of vocal units arranged in rhythmic and primarily isochronous patterns with species‐specific differences [5, 20, 21, 23, 52]. This suggests that while anatomical traits may influence phonatory stability and spectral properties, the temporal organization of primate vocalizations is shaped by a broader combination of anatomical, ecological, and social factors.

The growing body of research on singing primates reveals diversity in the temporal organization, both across and within species [53]. While some primates, such as titi monkeys, lar gibbons, and most crested gibbons, display simple isochronous patterns, other species show more rhythmic structures with multiple rhythmic categories [5, 20, 21, 22, 23]. Some singing primates produce rhythmic patterns that align with small‐integer ratios commonly found in human musical rhythm (e.g., 1:1, 2:1), suggesting the potential for shared mechanisms. However, not all species conform to these simple ratios, reflecting species‐specific temporal organization and vocal communication strategies [23], highlighting multiple evolutionary solutions for organizing a sequence of units over time. This is particularly evident in siamangs, which represent a unique example of a multimodal vocal production system. When the two vocal modalities are considered separately, their output shows a tendency toward isochrony, as observed in other gibbon species. However, when the emergent collective rhythm is considered, the resulting temporal organization comprises multiple rhythmic categories. As a consequence, the multimodal rhythmic patterns of siamangs cannot be fully described by traditional methodologies developed to test rhythmic categories based on small‐integer ratios typical of human music. This study highlights the need to reconsider what we consider rhythmicity and the methodological approach to assess it [43], to capture nonhuman forms of temporal structures that do not conform to the rules of human rhythm.

## Author Contributions

Conceptualization: L.L., A.R., and M.G. Data collection: A.S., S.P., G.R., M.T., and V.L.B. Methodology: L.L., A.R., and M.G. Formal analyses: L.L. Writing – original draft: L.L. Writing – review and editing: all authors. Visualization: L.L. and M.G. Supervision: V.I., A.R., and M.G.

L.L. and A.R. are funded by the European Union (ERC, TOHR, 101041885). The views and opinions expressed are those of the authors only and do not necessarily reflect those of the European Union or the European Research Council Executive Agency. Neither the European Union nor the granting authority can be held responsible for them. The Center for Music in the Brain is funded by the Danish National Research Foundation (DNRF 117). L.L. is also partially funded by Fondazione ZOOM.

## Conflicts of Interest

All authors declare no conflicts of interest.

## Supporting information




**Supplementary SuppMat**: nyas70273‐sup‐0001‐TableS1.docx

## Data Availability

The datasets used for the current study are available from the corresponding authors upon reasonable request.

## References

[1] L. S. Burchardt , P. Norton , O. Behr , C. Scharff , and M. Knörnschild , “General Isochronous Rhythm in Echolocation Calls and Social Vocalizations of the Bat *Saccopteryx Bilineata* ,” Royal Society Open Science 6, no. 1 (2019): 181076, 10.1098/rsos.181076. 30800360 PMC6366212

[2] A. R. Lameira , M. E. Hardus , A. Ravignani , T. Raimondi , and M. Gamba , “Recursive Self‐Embedded Vocal Motifs in Wild Orangutans,” Elife 12 (2023): RP88348, 10.7554/eLife.88348.3. PMC10945596 38252123

[3] C. De Gregorio , M. Gamba , and A. R. Lameira , “Third‐Order Self‐Embedded Vocal Motifs in Wild Orangutans, and the Selective Evolution of Recursion,” Annals of the New York Academy of Sciences 1549, no. 1 (2025): 219–229, 10.1111/nyas.15373. 40376956 PMC12309442

[4] V. Eleuteri , J. Van Der Werff , W. Wilhelm , et al., “Chimpanzee Drumming Shows Rhythmicity and Subspecies Variation,” Current Biology 35, no. 10 (2025): 2448–2456, 10.1016/j.cub.2025.04.019. 40347944

[5] T. Raimondi , G. Di Panfilo , M. Pasquali , et al., “Isochrony and Rhythmic Interaction in Ape Duetting,” Proceedings of the Royal Society B: Biological Sciences 290, no. 1990 (2023): 20222244, 10.1098/rspb.2022.2244. PMC9832542 36629119

[6] C. De Gregorio , D. Valente , T. Raimondi , et al., “Categorical Rhythms in a Singing Primate,” Current Biology 31, no. 20 (2021): R1379–R1380, 10.1016/j.cub.2021.09.032. 34699799

[7] V. Demartsev , M. Haddas‐Sasson , A. Ilany , L. Koren , and E. Geffen , “Male Rock Hyraxes That Maintain an Isochronous Song Rhythm Achieve Higher Reproductive Success,” Journal of Animal Ecology 92, no. 8 (2022): 1365–2656, 10.1111/1365-2656.13801. 36097377

[8] A. N. Osiecka , J. Fearey , A. Ravignani , and L. S. Burchardt , “Isochrony in Barks of Cape Fur Seal (*Arctocephalus pusillus pusillus*) Pups and Adults,” Ecology and Evolution 14, no. 3 (2024): e11085, 10.1002/ece3.11085. 38463637 PMC10920323

[9] K. Kocsis , D. Duengen , Y. Jadoul , and A. Ravignani , “Harbour Seals Use Rhythmic Percussive Signalling in Interaction and Display,” Animal Behaviour 207 (2024): 223–234, 10.1016/j.anbehav.2023.09.014.

[10] C. De Gregorio , I. Sondej , S. Previdi , F. Barbero , and L. P. Casacci , “Rhythmic Signaling of Ants and Butterflies with Varying Degrees of Myrmecophily,” Annals of the New York Academy of Sciences 1556, no. 1 (2026): e70223, 10.1111/nyas.70223. 41736302 PMC12932961

[11] M. Picciulin , M. Bolgan , and L. S. Burchardt , “Rhythmic Properties of Sciaena Umbra Calls across Space and Time in the Mediterranean Sea,” PLoS ONE 19, no. 2 (2024): e0295589, 10.1371/journal.pone.0295589. 38381755 PMC10881014

[12] M. Sebastianelli , S. M. Lukhele , S. Secomandi , et al., “A Genomic Basis of Vocal Rhythm in Birds,” Nature Communications 15, no. 1 (2024): 3095, 10.1038/s41467-024-47305-5. PMC11039653 38653976

[13] J. Xing , T. Sainburg , H. Taylor , and T. Q. Gentner , “Syntactic Modulation of Rhythm in Australian Pied Butcherbird Song,” Royal Society Open Science 9, no. 9 (2022): 220704, 10.1098/rsos.220704. 36177196 PMC9515642

[14] A. A. Ghazanfar , “Multisensory Vocal Communication in Primates and the Evolution of Rhythmic Speech,” Behavioral Ecology and Sociobiology 67, no. 9 (2013): 1441–1448, 10.1007/s00265-013-1491-z. PMC3821777 24222931

[15] M. Fröhlich , C. Sievers , S. W. Townsend , T. Gruber , and C. P. Van Schaik , “Multimodal Communication and Language Origins: Integrating Gestures and Vocalizations,” Biological Reviews 94, no. 5 (2019): 1809–1829, 10.1111/brv.12535. 31250542

[16] W. Pouw , S. Proksch , L. Drijvers , et al., “Multilevel Rhythms in Multimodal Communication,” Philosophical Transactions of the Royal Society B 376 (2021): 20200334, 10.1098/rstb.2020.0334. PMC8380971 34420378

[17] P. M. Narins , D. S. Grabul , K. K. Soma , P. Gaucher , and W. Hödl , “Cross‐Modal Integration in a Dart‐Poison Frog,” Proceedings of the National Academy of Sciences 102, no. 7 (2005): 2425–2429, 10.1073/pnas.0406407102. PMC548971 15677318

[18] J. A. C. Uy and R. J. Safran , “Variation in the Temporal and Spatial Use of Signals and Its Implications for Multimodal Communication,” Behavioral Ecology and Sociobiology 67, no. 9 (2013): 1499–1511, 10.1007/s00265-013-1492-y.

[19] C. De Gregorio , F. Carugati , D. Valente , et al., “Notes on a Tree: Reframing the Relevance of Primate Choruses, Duets, and Solo Songs,” Ethology Ecology & Evolution 34, no. 3 (2022): 205–219, 10.1080/03949370.2021.2015451.

[20] C. De Gregorio , P. Antonini , E. W. Heymann , and M. Gamba , “Isochrony in Titi Monkeys Duets: Social Context as a Proximate Cause of Duets' Rhythm and Regularity,” Proceedings of the Royal Society B: Biological Sciences 292, no. 2041 (2025): 20242805, 10.1098/rspb.2024.2805. PMC11836696 39968619

[21] C. De Gregorio , M. Maiolini , T. Raimondi , et al., “Isochrony as Ancestral Condition to Call and Song in a Primate,” Annals of the New York Academy of Sciences 1537, no. 1 (2024): 41–50, 10.1111/nyas.15151. 38925552

[22] C. De Gregorio , T. Raimondi , V. Bevilacqua , et al., “Isochronous Singing in 3 Crested Gibbon Species (*Nomascus* spp.),” Current Zoology 70, no. 3 (2023): 291–297, 10.1093/cz/zoad029. 39035758 PMC11255994

[23] H. Ma , Z. Wang , P. Han , et al., “Small Apes Adjust Rhythms to Facilitate Song Coordination,” Current Biology 34, no. 5 (2024): 935–945, 10.1016/j.cub.2023.12.071. 38266649

[24] T. Geissmann and M. Orgeldinger , “The Relationship between Duet Songs and Pair Bonds in Siamangs, *Hylobates Syndactylus* ,” Animal Behaviour 60, no. 6 (2000): 805–809, 10.1006/anbe.2000.1540. 11124879

[25] E. H. Haimoff , “Video Analysis of Siamang (*Hylobates syndactylus*) Songs,” Behaviour 76, no. 1–2 (1981): 128–151, 10.1163/156853981x00040.

[26] B. De Boer , “Acoustic Analysis of Primate Air Sacs and Their Effect on Vocalization,” Journal of the Acoustical Society of America 126, no. 6 (2009): 3329–3343, 10.1121/1.3257544. 20000947

[27] T. Riede , I. T. Tokuda , J. B. Munger , and S. L. Thomson , “Mammalian Laryngseal Air Sacs Add Variability to the Vocal Tract Impedance: Physical and Computational Modeling,” Journal of the Acoustical Society of America 124, no. 1 (2008): 634–647, 10.1121/1.2924125. 18647005 PMC2677336

[28] N. P. Todd and B. Merker , “Siamang Gibbons Exceed the Saccular Threshold: Intensity of the Song of *Hylobates Syndactylus* ,” Journal of the Acoustical Society of America 115, no. 6 (2004): 3077–3080, 10.1121/1.1736273. 15237832

[29] L. S. Burchardt , Y. Van De Sande , M. Kehy , M. Gamba , A. Ravignani , and W. Pouw , “A Toolkit for the Dynamic Study of Air Sacs in Siamang and Other Elastic Circular Structures,” PLOS Computational Biology 20, no. 6 (2024): e1012222, 10.1371/journal.pcbi.1012222. 38913743 PMC11226135

[30] T. Geissmann , “Gibbon Song and Human Music from an Evolutionary Perspective,” in N. L. Wallin , B. Merker , and S. Brown (Eds.) The Origins of Music (MIT Press, 2000), 103–123.

[31] T. Geissmann , “Duet Songs of the Siamang, *Hylobates Syndactylus*: I. Structure and Organisation,” Primate Report 56 (2000): 33–60. 10.1006/anbe.2000.1540 11124879

[32] D. J. Chivers , “Communication within and between Family Groups of Siamang (*Symphalangus syndactylus*),” Behaviour 57, no. 1–2 (1976): 116–135, 10.1163/156853976x00136.

[33] L. Morino , “Dominance Relationships among Siamang Males Living in Multimale Groups,” American Journal of Primatology 78, no. 3 (2016): 288–297, 10.1002/ajp.22365. 25598523

[34] S. M. Cheyne , “Behavioural Ecology of Gibbons (*Hylobates albibarbis*) in a Degraded Peat‐Swamp Forest,” in Indonesian Primates, ed. S. Gursky and J. Supriatna (Springer, 2010), 121–156, 10.1007/978-1-4419-1560-3_8.

[35] L. Morino , C. Pasquaretta , C. Sueur , and A. J. J. MacIntosh , “Communication Network Reflects Social Instability in a Wild Siamang (*Symphalangus syndactylus*) Population,” International Journal of Primatology 42, no. 4 (2021): 618–639, 10.1007/s10764-021-00227-1.

[36] J. D'Agostino , S. Spehar , A. Abdullah , and D. J. Clink , “Evidence for Vocal Flexibility in Wild Siamang (*Symphalangus syndactylus*) Ululating Scream Phrases,” International Journal of Primatology 44, no. 6 (2023): 1127–1148, 10.1007/s10764-023-00384-5.

[37] M. M. Haraway and E. G. Maples , “Flexibility in the Species‐Typical Songs of Gibbons,” Primates; Journal of Primatology 39, no. 1 (1998): 1–12, 10.1007/BF02557739.

[38] W. Pouw , M. Kehy , M. Gamba , and A. Ravignani , “Amplitude Increases of Vocalizations are Associated with Body Accelerations in Siamang (*Symphalangus syndactylus*).” International Journal of Comparative Psychology (2026 in press), 10.32942/X2C595.

[39] P. Boersma , “Praat, a System for Doing Phonetics by Computer,” Glot International 5 (2001): 341–345.

[40] G. Van Rossum and F. L. Drake Jr , “Python Reference Manual,” (Centrum voor Wiskunde en Informatica Amsterdam., 1995).

[41] R Core Team . (2022). R: A Language and Environment for Statistical Computing (R Foundation for Statistical Computing).

[42] T. C. Roeske , O. Tchernichovski , D. Poeppel , and N. Jacoby , “Categorical Rhythms Are Shared between Songbirds and Humans,” Current Biology 30, no. 18 (2020): 3544–3555, 10.1016/j.cub.2020.06.072. 32707062 PMC7511425

[43] Y. Jadoul , T. Tufarelli , C. Coissac , M. Gamba , and A. Ravignani , “ *Hidden Assumptions of Integer Ratio Analyses in Bioacoustics and Music* (Version 2),” arXiv (2025), 10.48550/ARXIV.2502.04464. PMC12645274 41043082

[44] A. Magnusson , H. Skaug , A. Nielsen , et al., “ Package ‘glmmTMB’. R Package Version 0.2.0 ,” (2019), https://cran.r‐hub.io/web/packages/glmmTMB/glmmTMB.pdf.

[45] D. Lüdecke , M. Ben‐Shachar , I. Patil , P. Waggoner , and D. Makowski , “performance: An R Package for Assessment, Comparison and Testing of Statistical Models,” Journal of Open Source Software 6, no. 60 (2021): 3139, 10.21105/joss.03139.

[46] A. J. Dobson , An Introduction to Generalized Linear Models, 2nd ed (Chapman & Hall/CRC, 2002).

[47] R. V. Lenth , P. Buerkner , I. Giné‐Vázquez , et al., “ Estimated Marginal Means, aka Least‐Squares Means ,” (2022), https://github.com/rvlenth/emmeans.

[48] J. V. Grabner , A. E. Kempf , A. M. N. Nederlof , J. M. Varkevisser , and M. J. Spierings , “ Rhythmic Roots: The Adaptive Functions of Vocal Isochrony and Its Role in Human Music and Language Evolution ,” (2025). 10.31820/pt.34.1.1 PMC7617685 40391320

[49] P. Norton and C. Scharff , ““Bird Song Metronomics”: Isochronous Organization of Zebra Finch Song Rhythm,” Frontiers in Neuroscience 10 (2016): 309, 10.3389/fnins.2016.00309. 27458334 PMC4934119

[50] H. Gouzoules , “When Less Is More in the Evolution of Language,” Science 377, no. 6607 (2022): 706–707, 10.1126/science.add6331. 35951706

[51] T. Nishimura , I. T. Tokuda , S. Miyachi , et al., “Evolutionary Loss of Complexity in Human Vocal Anatomy as an Adaptation for Speech,” Science 377, no. 6607 (2022): 760–763, 10.1126/science.abm1574. 35951711

[52] N. Jacoby and J. H. McDermott , “Integer Ratio Priors on Musical Rhythm Revealed Cross‐Culturally by Iterated Reproduction,” Current Biology 27, no. 3 (2017): 359–370, 10.1016/j.cub.2016.12.031. 28065607

[53] M. Gamba , C. D. Gregorio , D. Valente , et al., “Primate Rhythmic Categories Analyzed on an Individual Basis,” “Joint Conference on Language Evolution (JCoLE),” (2022).

