# Supplementary material for: Dual Sound Sources in Siamangs Generate Individually Rhythmic and Temporally Coordinated Vocal Emissions
Source: Ann N Y Acad Sci. 2026 Apr 21;1558:e70273. doi: 10.1111/nyas.70273 (PMC13099116; doi:10.1111/nyas.70273)
Supplement: Supplementary file 1 — Supplementary SuppMat: nyas70273‐sup‐0001‐TableS1.docx [file NYAS-1558-0-s001.docx]

**Supporting information**

**Table S1 -** Additional information about the dataset. For each individual, we specify the sex (male or female), and the zoo where the animal was recorded. We also report the recording duration, as the total amount of time the animal was singing, as well as the number recording in which the individual is singing alone (solo), with another individual (duets), or with more individuals (choruses).

| **ID** | **Zoo** | **Sex** | **Recording time** | **Solo** | **Duets** | **Choruses** |
| --- | --- | --- | --- | --- | --- | --- |
| Lady | Cincinnati | f | 286.4678 | 0 | 4 | 0 |
| Ohpy | Cincinnati | m | 308.8238 | 0 | 4 | 0 |
| Nina | Cornelle | f | 9660.3973 | 0 | 2 | 11 |
| Tomas | Cornelle | m | 10106.4503 | 0 | 2 | 11 |
| Puteri | Dubbo | f | 602.9909 | 0 | 10 | 0 |
| Saudara | Dubbo | m | 621.0506 | 0 | 10 | 0 |
| Berani | Houston | m | 166.265 | 0 | 2 | 0 |
| Jambi | Houston | f | 161.9024 | 0 | 2 | 0 |
| Eloise | Miami | f | 555.3736 | 0 | 6 | 0 |
| Unkie | Miami | m | 516.0695 | 0 | 6 | 0 |
| Franz | Ravenna | m | 72816.736 | 43 | 13 | 0 |
| Hans | Ravenna | m | 10127.8284 | 0 | 13 | 0 |
| Kiang | ZOOM | m | 29121.1012 | 2 | 22 | 2 |
| Queenia | ZOOM | f | 22705.8097 | 0 | 22 | 2 |

**On-integer and off-integer ratio ranges**. We centered the on-integer ratio ranges around three small-integer ratios: 1:1 (or 0.500, corresponding to isochrony), 1:9 (or 0.1), and 9:1 (or 0.9). For the 1:9 category, the boundaries of the on-integer ratio were 1:10.25 (or 0.098) and 1:9.75 (or 0.103). For isochrony, the boundaries were 1:2.25 (or 0.444) and 1:2.25 (or 0.555), while the 9:1 ratio fell between 1:9.75 (or 0.897) and 1:10.25 (or 0.902). For the 1:9 rhythmic category, the left-side off-integer ratio range was defined from 1:10.5 (or 0.095) to 1:10.25 (or 0.098), and the right-side range was from 1:9.75 (or 0.103) to 1:9.5 (or 0.105). For the 1:1 rhythmic category, the left-side off-integer ratio range extended from 1:2.5 (or 0.400) to 1:2.25 (or 0.444), and the right-side range was from 1:2.5 (or 0.556) to 1:2.25 (or 0.600). In the 9:1 rhythmic category, the left-side off-integer ratio range was defined from 1:9.5 (or 0.895) to 1:9.75 (or 0.897), while the right-side range extended from 1:10.25 (or 0.902) to 1:10.5 (or 0.905).
